# Design of self-emulsifying oral delivery systems for semaglutide: reverse micelles versus hydrophobic ion pairs

**DOI:** 10.1007/s13346-024-01729-0

**Published:** 2024-10-19

**Authors:** Matthias Sandmeier, Fabrizio Ricci, Dennis To, Sera Lindner, Daniel Stengel, Michaela Schifferle, Saadet Koz, Andreas Bernkop-Schnürch

**Affiliations:** 1 Department of Pharmaceutical Technology, Institute of Pharmacy, Center for Chemistry and Biomedicine, University of Innsbruck, Innsbruck, 6020 Austria; 2 Thiomatrix Forschungs- und Beratungs GmbH, Trientlgasse 65, Innsbruck, 6020 Austria

**Keywords:** Oral peptide delivery, Self-emulsifying drug delivery systems, Reverse micelles, Semaglutide, Hydrophobic ion pairing, Lipid-based nanocarriers

## Abstract

It was the aim of this study to evaluate the potential of reverse micelles (RM) and hydrophobic ion pairs (HIP) for incorporation of semaglutide into self-emulsifying oral drug delivery systems. Reverse micelles loaded with semaglutide were formed with a cationic (ethyl lauroyl arginate, ELA) and an anionic surfactant (docusate, DOC), whereas HIP were formed between semaglutide and ELA. Maximum solubility of the peptide and the rate of dissolution was evaluated in various lipophilic phases (glycerol monocaprylocaprate:caprylic acid 1:4 (m/m), glycerol monolinoleate:caprylic acid 1:4 (m/m) and glycerol monocaprylocaprate:glycerol monolinoleate 1:4 (m/m)). Self-emulsifying drug delivery systems (SEDDS) loaded with RM and HIP were characterized regarding size distribution, zeta potential, cytocompatibility and Caco-2 permeability. Droplet sizes between 50 and 300 nm with polydispersity index (PDI) around 0.3 and zeta potentials between − 45 mV (RM_DOC_) and 36 mV (RM_ELA_) were obtained. RM provided an almost 2-fold higher lipophilicity of semaglutide than HIP resulting in a 4.2-fold higher payload of SEDDS compared to HIP. SEDDS containing RM or HIP showed high cytocompatibilities with a cell survival above 75% for concentrations up to 0.1% on Caco-2 cells and acceptable hemolytic activity. Permeation studies across Caco-2 monolayer revealed an at least 2-fold increase in permeability of semaglutide for the developed formulations.

**Supplementary Information:**

The online version contains supplementary material available at 10.1007/s13346-024-01729-0.

## Statement of Significance

Hydrophilicity of semaglutide limits its absorption after oral administration by poor membrane permeability. Lipid-based delivery systems show an enhanced permeation of the lipid bilayer, thus improving the oral bioavailability of drugs. However, the hydrophilic character of semaglutide restricts direct incorporation into lipid-based delivery systems. Two different methods to enhance the lipophilicity of semaglutide enabling the incorporation into self-emulsifying drug delivery systems are described, yielding high semaglutide payload and an increased Caco-2 permeation of this GLP-1 analogue. These methods pave the way for more efficient oral delivery of semaglutide.

**Graphical abstract:**

**Supplementary Information:**

The online version contains supplementary material available at 10.1007/s13346-024-01729-0.

## Graphical abstract

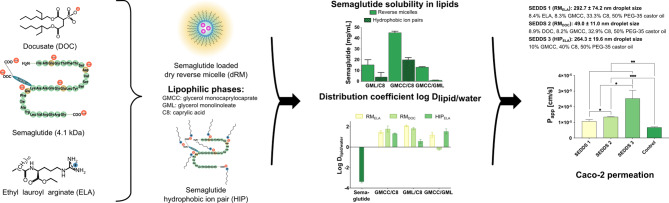

## Introduction

Administration of therapeutic (poly)peptides is still widely limited to the parenteral route even though oral administration is both favored from a patient as well as from a commercial point of view [1]. So far mostly endocrine disorders and in particular diabetes have been targeted by oral (poly)peptide drug delivery [2, 3]. Administration of (poly)peptides such as insulin or GLP-1 analogues via the oral pathway, however, is challenging because of the inactivation of these therapeutic agents under harsh pH conditions, degradation by proteolytic enzymes, thiol/disulfide exchange reactions with mucus substructures and last but not least poor membrane permeability due to their size above 1 kDa and hydrophilic character [4, 5]. Among different strategies that have been developed to overcome these barriers, lipid-based nanocarriers and in particular self-emulsifying drug delivery systems (SEDDS) turned out most promising. In order to incorporate antidiabetic (poly)peptides into SEDDS, enhancement of lipophilicity is essential. By the formation of hydrophobic complexes utilizing charged or ionizable surfactants so called hydrophobic ion pairs (HIP) with ionizable functional groups of the peptide can be formed [6]. For insulin, ion pairing resulted in a tremendous increase in lipophilicity of this anti-diabetic drug resulting in an up to 8-fold improvement in oral bioavailability and a significantly decreased blood glucose level [7, 8]. For the GLP-1 analogue exenatide, enhanced lipophilicity of HIP formed with octadecyl sulfate or tetraheptylammonium was observed resulting also in an improved oral bioavailability [9–11]. The limited number of charged or ionizable functional groups on these (poly)peptides, however, predetermines the number of possible interactions with hydrophobic counter ions resulting in low drug payloads or premature release from SEDDS [12, 13]. A strategy recently developed by Jörgensen et al. addresses this shortcoming by the formation of reverse micelles in that even uncharged (poly)peptide drugs can be incorporated [14]. The self-assembly of polar head groups of surfactants in a lipophilic phase leads to the formation of hydrophilic cavities in that (poly)peptides can be encapsulated. The lipophilic phase containing (poly)peptides can subsequently be formulated to SEDDS [14, 15]. So far, however, detailed investigations on the formation of reverse micelles in particular with GLP-1 analogues as well as a direct comparison with HIP are still missing.

It was therefore the aim of this study to evaluate the potential of reverse micelles versus HIP for the formation of lipophilic complexes to be incorporated into SEDDS. Among GLP-1 analogues semaglutide was chosen as model drug, since a marketed oral formulation (Rybelsus^®^) is already available [16, 17]. Besides its use for treatment of type 2 diabetes, this peptide has recently gained huge attention as anti-obesity drug [18, 19]. Even though oral semaglutide showed impressive efficacy in various trials [20, 21], the overall bioavailability is just around 1%, indicating much room for improvements to reduce the cost per dose [16, 19]. Best performing RM were incorporated into SEDDS and compared to HIP formed with the already as food additive approved surfactant ethyl lauroyl arginate. SEDDS were characterized regarding droplet size, PDI, zeta potential, payload, self-emulsifying properties, semaglutide release and cytotoxicity. Furthermore, Caco-2 permeation of SEDDS loaded with RM or HIP were performed and compared to unformulated semaglutide.

## Materials and methods

### Materials

Semaglutide was purchased from Mobelbiochem (Hangzhou, China). Ethyl lauroyl arginate HCl was received from Connect Chemicals (Vimercate, Italy). Docusate sodium, bovine serum albumin and trifluoroacetic acid were purchased from Thermo Fisher Scientific (Vienna, Austria). Labrafac MC 60 and Maisine CC were kindly provided by Gattefossé (Saint-Priest, France). Kolliphor EL was received from BASF (Ludwigshafen, Germany). All solvents (HPLC grade) and all other chemicals were obtained from Sigma-Aldrich (Vienna, Austria).

### Methods

#### HPLC quantification of peptides

An HPLC System Merck, LaChrome Elite for Hitachi consisting of a 5110 pump, an L-2200 autosampler, an L-2350 column oven and an L-2400 A UV detector was used to quantify semaglutide and reference (poly)peptides. The HPLC methods were adapted from previously published quantification methods [14, 22, 23]. Specifications for the methods applied are listed in Table 1 and the corresponding gradients in Table S1 to Table S3. The HPLC method resulted in adequate peak shapes (Supporting info (SI) Figure S1 to Figure S4) and calibration curves with sufficient linearity (R^2^ ≥ 0.990).

**Table 1:** HPLC settings for quantification of semaglutide and reference (poly)peptides

Settings	Semaglutide	Bovine serum albumine & Lysozyme	Colistin
Stationary phase	WATERS Xbridge^®^ C18 (5 μm, 4.6 × 250 mm)	WATERS XBridge^®^ Protein BEH C4 (300 Å, 3.5 μm, 4.6 × 50 mm)	Machery-Nagel Nucleosil^®^ C18 (5 μm, 4.6 × 50 mm)
Duration	10 min	12 min	10 min
Column temperature	40 °C	40 °C	40 °C
Autosampler temperature	10 °C	10 °C	10 °C
Mobile phase	Water: ACN (50:50, v/v) with 0.1% TFA	Water: ACN (80:20, v/v) with 0.1% TFA	Water: ACN (70:30, v/v) with 0.1% TFA
Injection volume	20 µL	20 µL	20 µL
Flow rate (ml/min)	1 mL/min	1 mL/min	1 mL/min
Type	Gradient	Gradient	Gradient
Detection (nm)	210 nm	210 nm	210 nm

#### Formation of reverse micelles

The following oily mixtures were investigated for reverse micelle formation: glycerol monocaprylocaprate (GMCC):caprylic acid (C8) in a ratio of 1:4 (m/m), glycerol monolinoleate (GML):caprylic acid (C8) in a ratio of 1:4 (m/m), glycerol monocaprylocaprate (GMCC):glycerol monolinoleate (GML) in a ratio of 1:4 (m/m). Reverse critical micelle concentration (rCMC) in three different oil mixtures was determined by a previously described method [14]. In brief, empty RMs were formed by dissolving ELA or DOC in oily mixtures as described above in final concentrations of 0 mM to 300 mM. To 500 µL of these solutions 0.5 mg of 7,7,8,8-tetracyanodimethane (TCNQ) were added and samples were vigorously agitated on a thermomixer (Eppendorf, Germany) at 1200 rpm for 24 h at ambient temperature. Thereafter, undissolved TCNQ was removed from samples by centrifugation at 12,045 RCF for 10 min. Absorbance was measured at 480 nm utilizing a microplate reader (TECAN Infinite 200 Pro M Nano, Austria). rCMC was calculated from the inflection point of the plot of absorbance against the surfactant concentration.

rCMC was confirmed by the maximum water uptake of empty reverse micelles. Water was incrementally added to 500 µL of surfactant-oil solutions in aliquots of 2 µL until cloudiness, milky appearance or phase separation was observed.

(Poly)peptide loaded RM were prepared utilizing 200 mM of surfactants dissolved within the oily phase (GML/C8 (1:4; m/m), GMCC/C8 (1:4; m/m) and GMCC/GML (1:4; m/m)) in order to guarantee formation of reverse micelles. Loaded reverse micelles were prepared by the addition of semaglutide to surfactant-oil solutions (200 mM ELA or 200 mM DOC) resulting in a final concentration of 50 mg/mL. The mixtures were vortexed and treated with ultrasound for 30 min before incubation at 1200 rpm at room temperature (RT). Formation of reverse micelles by dry addition method, describing the direct addition of the API in powdered form to lipophilic solvents without use of any additional solvents [24], was investigated for semaglutide and three further (poly)peptides for comparison. In that regard, semaglutide with a molecular weight of 4.1 kDa was compared to colistin representing the smallest investigated peptide (1.1 kDa). In addition, two larger polypeptides, lysozyme (LYS, 14.4 kDa) and bovine serum albumin (BSA, 66.4 kDa) were investigated. At predetermined time points the dissolved (poly)peptide in supernatant was quantified by HPLC after centrifugation at 12,045 RCF for 10 min.

#### Hydrophobic ion pairing studies with ethyl lauroyl arginate

Hydrophobic ion pairs were formed in order to increase lipophilicity of semaglutide according to previous studies [25–27]. The peptide was dissolved in 0.001 M NaOH in a concentration of 10 mg/ml to obtain a negative net charge. Ethyl lauroyl arginate, as a cationic counter ion was dissolved in demineralized water. Equal amounts (500 µL: 500 µL) of peptide and surfactant solution were combined corresponding to a charge ratio of 1:1 (peptide:surfactant). Samples were agitated at 400 rpm for 2 h on a thermomixer (Eppendorf, Austria) at ambient temperature. Precipitates were separated from the solution by centrifugation at 12,045 RCF for 10 min (Minispin, Eppendorf, Austria). Precipitation efficiency was determined according to the following equation:

1 $$\:Precipitation\:efficiency\:\left[\%\right]=100*(1-\frac{{c}_{Peptide\:after\:HIP}}{{c}_{Peptide\:before\:HIP}})$$

Hydrophobic ion pairs were washed with 0.001 M NaOH, dried utilizing a SpeedVac and stored at -20 °C until further use. Formation of ion pairs between ethyl lauroyl arginate and semaglutide were confirmed by FTIR analysis of 3 mg of the corresponding powders using a Bruker ALPHA FTIR (Billerica, MA, USA).

#### Determination of log D(n-octanol/water)

Increase in lipophilicity of formed complexes was characterized by determination of the log D_n-octanol/water_ as previously described by Phan et al. [26]. Semaglutide loaded RM were formed by the addition of semaglutide powder to a 200 mM surfactant solution (200 mM ELA or 200 mM DOC) in n-octanol resulting in a final concentration of 50 mg/mL. The mixtures were vortexed and treated with ultrasound for 30 min before incubation at 1200 rpm at room temperature (RT) for 48 h. After centrifugation at 12,045 RCF for 10 min, 200 µL of the supernatant were underlaid with equal volume of water and incubated at 300 rpm for 24 h at room temperature. Samples were centrifuged for 10 min at 12,045 RCF, aliquots of each phase were withdrawn and peptide content was quantified via HPLC. HIP complexes corresponding to 2 mg of semaglutide were dispersed in 200 µL of n-octanol and treated with ultrasound (Elma, Germany) for 30 min. Thereafter, 200 µL of water were added and the mixture was incubated at 300 rpm for 24 h at room temperature. Samples were centrifuged at 12,045 RCF for 10 min, aliquots of each phase were withdrawn and peptide content was quantified via HPLC. Log D_n-octanol/water_ was calculated according to:

2 $$\:{log\:D}_{n-octanol/water}=log\frac{{c}_{peptide\:in\:n-octanol\:phase}}{{c}_{peptide\:in\:water\:phase}},$$

where $\:{c}_{peptide\:in\:n-octanol\:phase}$ corresponds to peptide concentration in the n-octanol phase after incubation and $\:{c}_{peptide\:in\:water}$ to peptide concentration in the water phase after incubation.

#### Determination of log D(lipid phase/water)

Retention of drug within lipid phase after emulsification is crucial for the active ingredient to reach the absorption membrane in intact form. Thus, high log D_lipid phase/water_ values are substantial for lipophilic complexes of semaglutide. In brief, 200 µL of peptide loaded RM or HIP complexes corresponding to 2 mg of semaglutide in the lipid phases GML/C8 (1:4; m/m), GMCC/C8 (1:4; m/m) and GMCC/C8 (1:4; m/m) were underlaid with 200 µL of demineralized water and incubated at 300 rpm on a thermomixer for 24 h. In the following samples were centrifuged at 12,045 RCF for 10 min and 100 µL aliquots from both phases were withdrawn and the amount of peptide was quantified by HPLC. The distribution coefficient was calculated according to Eq. 3:

3 $$\:{log\:D}_{lipid\:phase/water}=log\frac{{c}_{peptide\:in\:lipid\:phase}}{{c}_{peptide\:in\:water}},$$

where $\:{c}_{peptide\:in\:lipid\:phase}$ corresponds to peptide concentration in the lipophilic phase after incubation and $\:{c}_{peptide\:in\:water}$ to peptide concentration in the water phase after incubation. Log D_lipid phase/water_ values for reference (poly)peptides were determined in the same manner.

#### Development of SEDDS

**Fig. 1:**
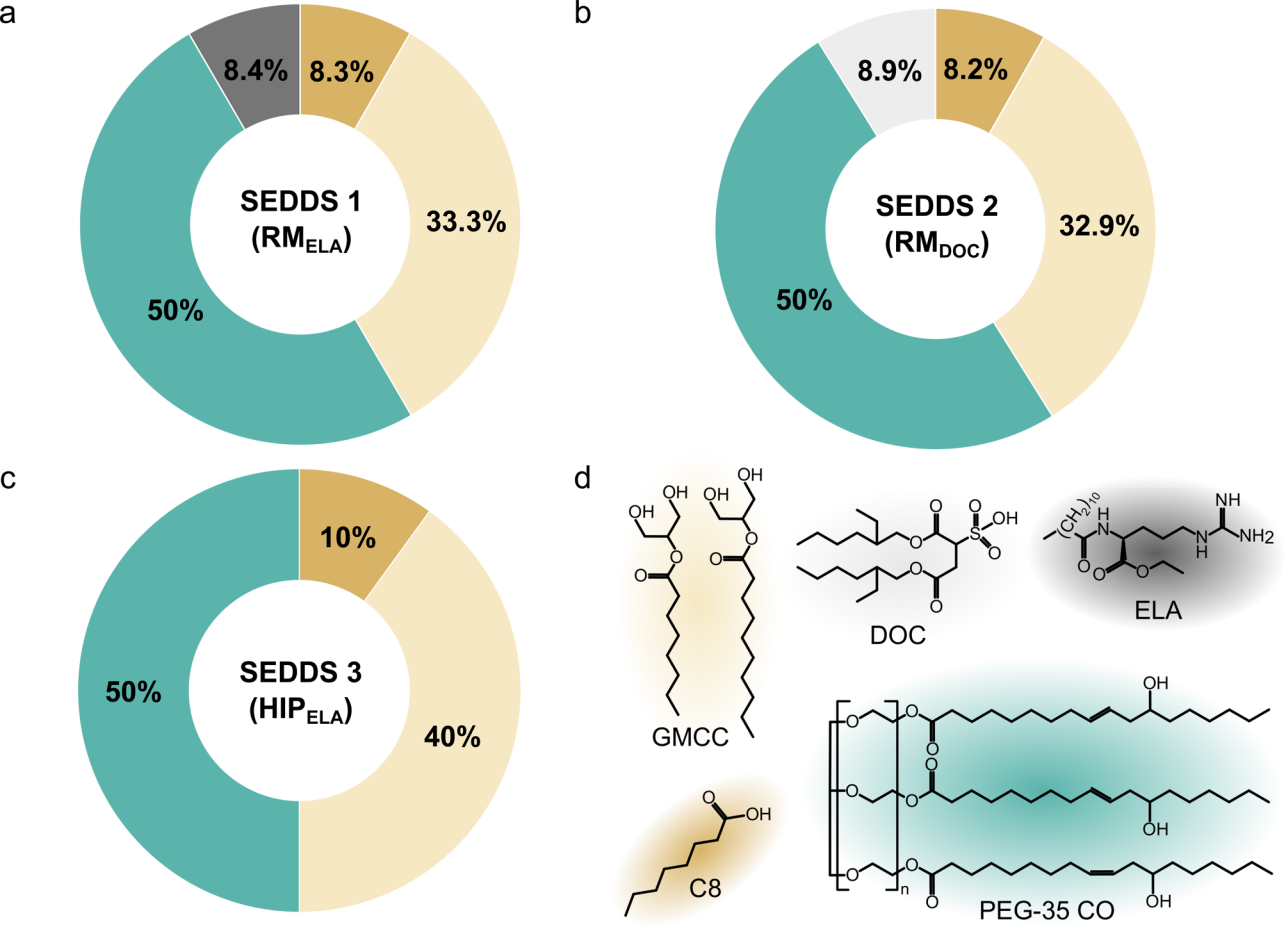
**a – c** Composition of SEDDS formulations and **d** chemical structures of excipients. C8: caprylic acid, DOC: docusate, ELA: ethyl lauroyl arginate, GMCC: glycerol monocaprylocaprate, PEG-35 CO: PEG-35 castor oil

SEDDS preconcentrates were prepared by mixing corresponding amount of PEG-35 castor oil to loaded RM mixtures using a vortex mixer and ultrasonication (Table 2; Fig. 1). Homogeneity of RM and PEG-35 castor oil were visually evaluated. In contrast, HIP containing SEDDS were prepared by mixing of excipients utilizing a vortex mixer and ultrasonication until a clear homogeneous solution was obtained. HIP were loaded in SEDDS by incubation of complexes corresponding to 2 mg of semaglutide with SEDDS in a concentration of 50 mg/mL for 30 min in an ultrasonic bath and incubation for 48 h under shaking conditions (1200 rpm, RT). After centrifugation at 12,045 RCF for 10 min, clear supernatant, containing dissolved HIP, was utilized for further experiments. The encapsulation efficiency (EE) of formulations was calculated according to the equation below:

4 $$\:EE\:\%=\frac{Semaglutide\:in\:SEDDS\:\left[\frac{mg}{mL}\right]}{Maximum\:semaglutide\:payload*\frac{\%\:of\:RM\:in\:SEDDS}{100}}*100,$$

where the $\:Maximum\:semaglutide\:payload$ is based on the utilized semaglutide for preparation of reverse micelles and $\:\text{\%}\:of\:RM\:in\:SEDDS$ the combined amount of GMCC, C8 and ELA or DOC in the formulation. Formulations were diluted 1:100 (v/v) with purified water or fasted-state simulated gastric fluid (FaSSGF, SI Table S4) and droplet size, polydispersity index (PDI) and zeta potential (NanoBrook 90 Plus PALS; Brookhaven) were determined directly after emulsification as well as after incubation at 300 rpm for 4 h at 37 °C.      

Time required for emulsification of preconcentrates diluted 1:100 (v/v) with demineralized water was determined. Hence, preheated demineralized water (37 °C) was added to 50 µL of preconcentrates placed into a clear glass vial. Samples were gently agitated with a magnetic bar at 300 rpm in an incubator chamber set at 37 °C to simulate in vivo emulsification by gastrointestinal motion. Assessment of the required time to complete emulsification was performed visually.

**Table 2:** Composition of SEDDS (%. m/m)

Formulation	DOC	ELA	GMCC	C8	EL
SEDDS 1 (RM_ELA_)		8.4	8.3	33.3	50
SEDDS 2 (RM_DOC_)	8.9		8.2	32.9	50
SEDDS 3 (HIP_ELA_)			10	40	50

ELA: ethyl lauroyl arginate, C8: caprylic acid, DOC: docusate sodium, EL: PEG-35 castor oil, GMCC: glycerol monocaprylocaprate

#### Characterization of SEDDS

Preconcentrates loaded with semaglutide were diluted 1:100 (v/v) with demineralized water and agitated for 4 h at 300 rpm and 37 °C. Resulting emulsions were analyzed regarding droplet size, PDI and zeta potential immediately after emulsification and after 4 h of incubation by dynamic light scattering (DLS, NanoBrook 90 Plus PALS; Brookhaven).

Payload of SEDDS was determined after mixing of semaglutide loaded RM with PEG-35 castor oil and treatment of samples with ultrasound for 30 min at a maximum of 40 °C. Preconcentrates were centrifuged at 12,045 RCF for 10 min and the concentration of semaglutide in the supernatant was quantified by HPLC.

In order to prove retention of semaglutide within SEDDS emulsified in biomimetic media, release of semaglutide from SEDDS was determined using a diffusion membrane method [28]. Dialysis tubes (Spectra/Por^®^ Biotech CE) with a cut-off of 100 kDa were filled with SEDDS dispersed in phosphate buffer pH 6.8 1:100 (v/v) to a total volume of 1 mL in the dialysis tube. The peptide solutions were dialyzed in a 50 mL Falcon Tube against 15 mL of phosphate buffer pH 6.8 at 37 °C. Gastrointestinal movement was simulated by agitation at 300 rpm in a thermomixer. At predetermined time points 100 µL aliquots were withdrawn from the release medium and replaced by the same volume of fresh release medium. Free peptide in release medium ($\:{c}_{Release\:medium}$) was quantified via HPLC. As reference, 1 mL of semaglutide dissolved in phosphate buffer pH 6.8 (20 mg/mL) in a dialysis tube, indicating potential membrane interaction, was incubated in the same manner as SEDDS. Log D_SEDDS/Release medium_ was calculated using the following equation:

5 $$\:{\text{log}\text{D}}_{\text{SEDDS/Release medium}}\text{=}\text{log}\frac{{c}_{SEDDS}}{{c}_{Release\:medium}},$$

where $\:{c}_{SEDDS}$ corresponds to peptide concentration in prepared SEDDS.

#### Toxicological studies

Cell studies were performed on Caco-2 cell line (ECACC 86010202), previously described as suitable model for the intestinal epithelial membrane [29]. Thus, cells were seeded in a density of 5 × 10^5^ Caco-2 cells/mL in a 96-well plate. Minimal essential medium (MEM) containing 10% (v/v) heat inactivated fetal calf serum (FCS) and penicillin/streptomycin solution (100 units in a concentration of 0.1 mg/L) was utilized as nutrition medium. Cells were incubated for 120 h at 95% humidity and 37 °C in an atmosphere of 5% CO_2_ to allow formation of a monolayer.

Compatibility of SEDDS on Caco-2 cells was evaluated [30]. To that end, emulsified SEDDS ranging from 0.01 to 1% (v/v) in iso-osmolar, sterile 5% (w/v) glucose-20 mM HEPES buffer pH 7.4 were prepared. MEM on cell layer was discarded and replaced with 100 µL of emulsified SEDDS in sterile 5% (w/v) glucose-20 mM HEPES buffer pH 7.4 for the initiation of the experiment. Samples were removed after an incubation period of 4 h at 37 °C, and cells were washed twice using 5% (w/v) glucose-20 mM HEPES buffer to remove SEDDS remaining on the surface of cells. Thereafter, cells were incubated with 150 µL of a 0.1% (m/v) resazurin solution in indicator-free MEM for 2 h. The fluorescence of 100 µL of supernatant was analyzed at an excitation wavelength of 540 nm and an emission wavelength of 590 nm using a microplate reader (TECAN Infinite 200 Pro M Nano, Austria). Sterile glucose-HEPES buffer pH 7.4 and ethanol 96% were utilized as negative and positive control, respectively. The fraction of living cells was calculated according to *Eq. 6*:

6 $$\begin{aligned}& Cell\:viability\left[ \%  \right] \\ & \quad  = \frac{{Fluorescenc{e_{Sample}} - Fluorescenc{e_{negative}}}}{{Fluorescenc{e_{positive}} - Fluorescenc{e_{negative}}}}*100. \\ \end{aligned} $$

High amounts of surfactants and especially cationic nanoparticles are known to bear high membrane disruption potential. Hence, the hemolytic activity of SEDDS on human erythrocytes was investigated. Thus, human erythrocyte concentrate, obtained from Tirol Kliniken GmbH (Innsbruck, Austria), was suspended in a ratio of 1:200 (v/v) with sterile 5% (w/v) glucose 20 mM HEPES buffer pH 7.4 [31]. SEDDS preconcentrates were emulsified in concentrations of 0.2%, 1.0% and 2.0% (v/v) in 20 mM HEPES buffer pH 7.4. 500 µL of erythrocyte suspension were incubated with equal volume of emulsified SEDDS at 150 rpm at 37 °C for 4 h. Aliquots of the mixture were withdrawn and centrifuged at 1780 RCF for 1 min. Absorbance of the supernatant was determined at 415 nm using a microplate reader (TECAN Infinite 200 Pro M Nano, Austria). As negative and positive control 5% (w/v) glucose-20 mM HEPES buffer pH 7.4 and 0.1% Triton X solution (v/v) were used, respectively. The hemolytic acitivity was quantified according to:

7 $$\begin{aligned}& Hemolytic\:acitvity\:\left[ \%  \right] \\ & \quad  = \frac{{Absorbtio{n_{Sample}} - Absorptio{n_{negative}}}}{{Absorptio{n_{positive}} - Absorptio{n_{negative}}}}*100. \\ \end{aligned} $$

#### Caco-2 permeation studies

The ability of native semaglutide as well as semaglutide-RM loaded SEDDS and SEDDS containing HIP to permeate the intestinal epithelium was investigated using Caco-2 cells. Caco-2 cell line was seeded on a 24-well Transwell filters plate at a cell density of 1.25 × 10^5^ cells/cm^2^. Cells were cultivated for 21 days replacing the media every second day. On the day of the experiment, cells were washed twice using sterile HBS buffer pH 6.8. To start the experiment after the equilibration period, the buffer was removed and replaced with 100 µL of either native semaglutide solution in sterile HBS buffer pH 6.8 or semaglutide-RMs loaded SEDDS diluted to a concentration of 0.05% (v/v) with sterile HBS buffer pH 6.8. Sterile HBS buffer (500 µL) was added to basolateral side. At predetermined time points, 100 µL were withdrawn from basolateral chambers and replaced with prewarmed HBS. The amount of semaglutide in the basolateral chamber was measured through ELISA (KRIBIOLISA™ Semaglutide ELISA, Krishgen BioSystems, US). Transepithelial electric resistance (TEER) in a range of 1200–3300 ohm/cm^2^ were measured ensuring integrity of cell monolayers (Evom2, World Precision Instrument Inc., USA) [32].

#### Statistical data analyses

Statistical data analyses were performed using Student’s t-test between two independent means. Hereby, p-values < 0.05 were considered significant (*), *p* < 0.01 as very significant (**) and *p* < 0.001 as highly significant (***). Results are means of at least three experiments ± standard deviation (SD).

## Results and discussion

### Formation of reverse micelles

**Fig. 2:**
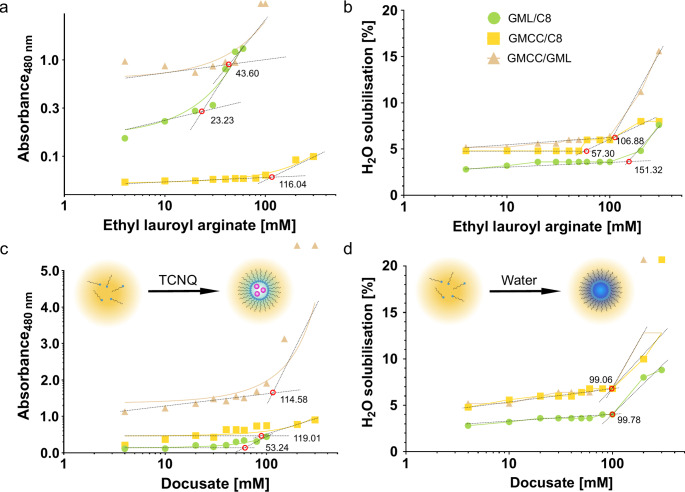
**a** Plots of absorbance of TCNQ solubilized in oil mixtures containing ethyl lauroyl arginate at a wavelength of 480 nm and **b** plots of water solubilization as a function of surfactant concentration. **c** Plots of absorbance of TCNQ solubilized in oil mixtures containing docusate at a wavelength of 480 nm with schematic drawing of the working principle for rCMC determination by TCNQ method. **d** Plots of water solubilization as a function of surfactant concentration with schematic drawing of the working principle for rCMC determination by water solubilization method. Red circles and numbers at the intersection points indicating rCMC

As for the incorporation of polar molecules like semaglutide a micellar structure is needed, minimal concentrations of the surfactants resulting in reverse micelles were determined. The formation of micelles is characterized by an increase in absorbance indicating the incorporation of the polar marker TCNQ [14]. GML/C8 containing 200 mM ethyl lauroyl arginate showed a markedly lower rCMC compared to the water uptake study (Fig. 2). This might be a result of the low HLB values of both lipids forming the lipid phase enabling a comparatively low water uptake. Nevertheless, all formulations exhibited rCMCs around 100 mM irrespectively of the method applied. Furthermore, no pronounced difference could be shown between docusate and ethyl lauroyl arginate. The obtained rCMCs are roughly in agreement with previous results for docusate in nonpolar solvents even though the rCMC is assumed to be specific for each ternary system [14, 33, 34].

**Fig. 3:**
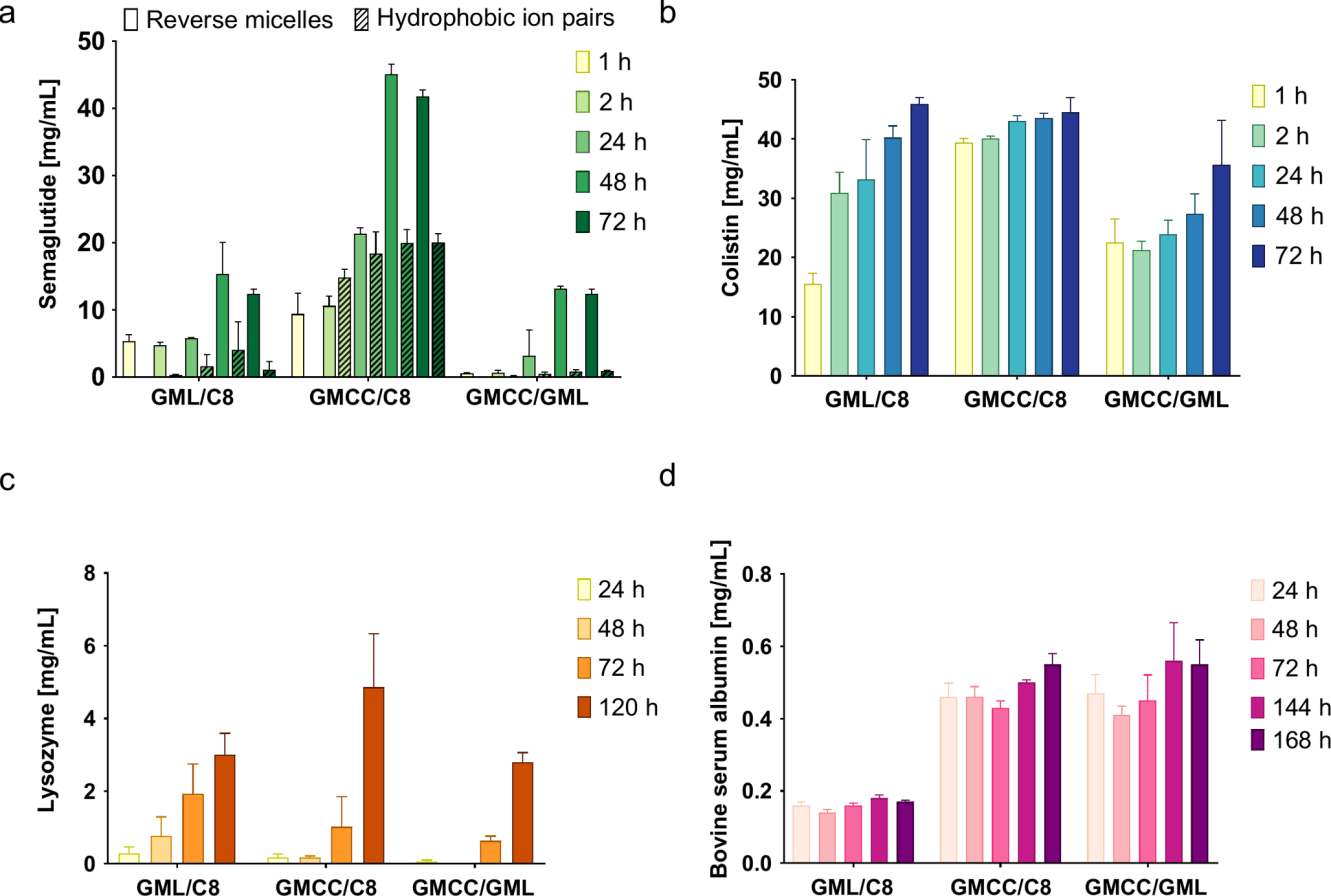
Solubility of **a** semaglutide, **b** 1.1 kDa reference peptide, **c** 14.4 kDa reference polypeptide and **d** 66.4 kDa reference polypeptide within reverse micelles utilizing different incubation times. Indicated values are means (n ≥ 3) ± SD

Dissolution of peptides within reverse micelles utilizing dry addition method was shown to take approximately 24 h in order to achieve solubility of semaglutide above 10 mg/mL. Semaglutide exhibited increase in solubility over time with a maximum of almost 100% after 48 h to 72 h (Fig. 3a). In RM an at least 2-fold higher solubility of semaglutide was observed in the best performing lipid phase (glycerol monocaprylocaprate: caprylic acid 1:4 (m/m)) in comparison to HIP even though semaglutide solubility was similar within the first 24 h of incubation. In general, for HIP maximum solubility of semaglutide was achieved within around 24 h whereas maximum solubility in RM was only observed within 48 h. This suggests, that encapsulation of semaglutide within the polar cavities of RM is comparatively slower than solubilization of already lipidized semaglutide complexes such as HIP. A similar trend was observed for encapsulation of a 1.1 kDa reference peptide in RM (Fig. 3b). Furthermore, the solubility of the 14.4 kDa reference polypeptide was about 10-times lower compared to semaglutide with a pronounced increase in solubility only after 72 h (Fig. 3c). The low incorporation of polypeptides into reverse micelles suggests a correlation between molecular size and the encapsulation of polypeptides in reverse micelles. This could be further confirmed by the 66.4 kDa reference polypeptide resulting in solubilities below 1 mg/mL even after prolonged incubation time (Fig. 3d). A similar dependence of (poly)peptide size affecting the incorporation of larger (poly)peptides into lipid-based delivery systems has been previously reported for hydrophobic ion pairs [23]. No general trend of the influence of isoelectric point (IEP) of (poly)peptides on maximum solubility in RM could be observed as both semaglutide (IEP approx. 5.0 [35]) and the 1.1 kDa reference peptide (IEP 5.0 [36]) showed high solubility whereas both larger polypeptides (14.4 kDa polypeptide, IEP 11.0 [37] and 66.4 kDa polypeptide, IEP 5.0 [38]) showed low solubility. In general, highest (poly)peptide solubilities were obtained in glycerol monocaprylocaprate:caprylic acid in a ratio of 1:4 (m/m).

### Hydrophobic ion pairing studies with ethyl lauroyl arginate

**Fig. 4:**
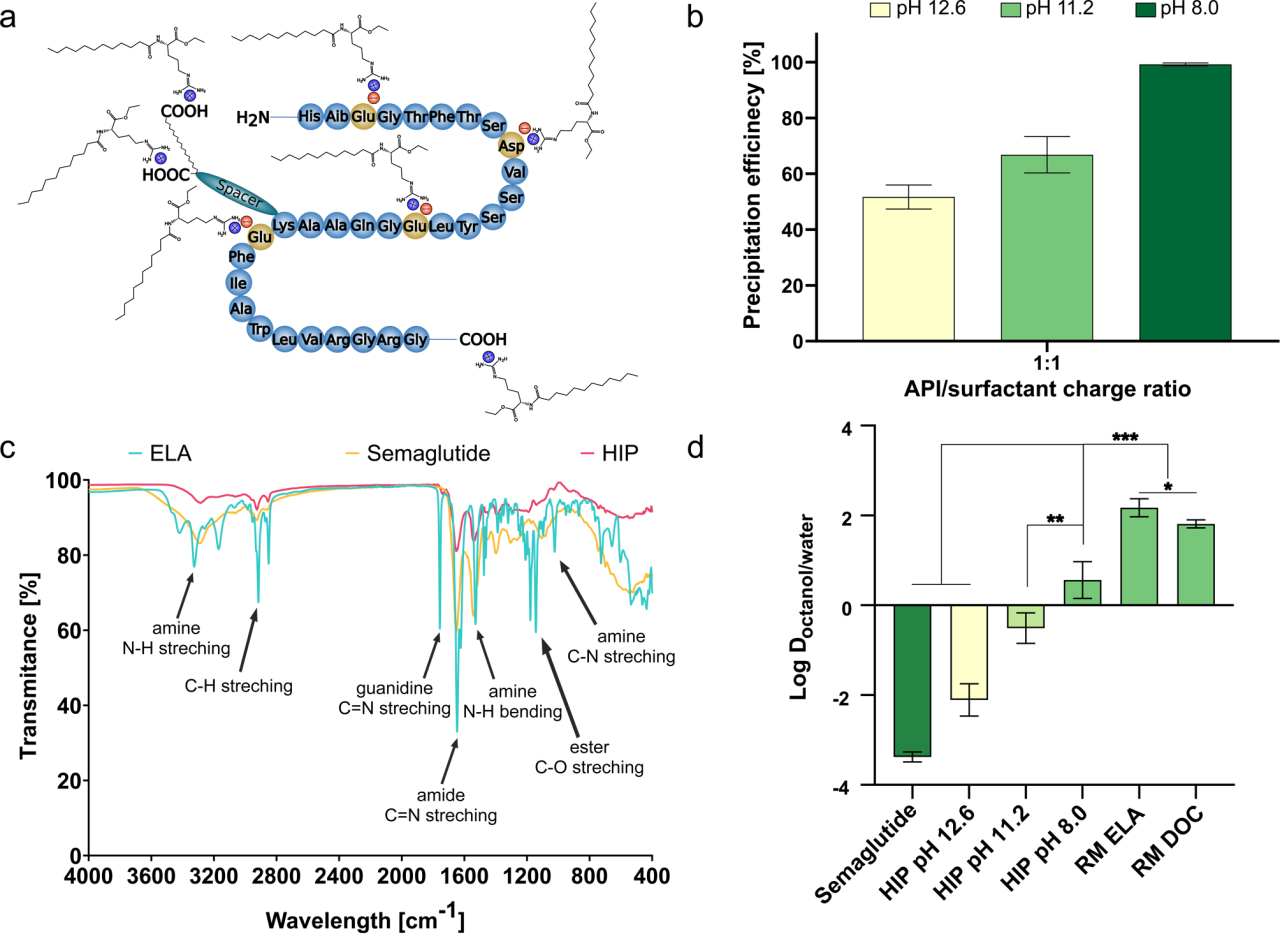
**a** Illustration of semaglutide: ethyl lauroyl arginate (ELA) hydrophobic ion pair (HIP). **b** Precipitation efficiency of semaglutide: ELA HIP with the peptide dissolved in NaOH solutions of different concentrations. **c** Fourier transformed infrared (FTIR) spectra of native ELA, native semaglutide and semaglutide: ELA HIP. **d** Distribution coefficient log D_n-octanol/water_ of native semaglutide and semaglutide: ELA HIP with the peptide dissolved in NaOH solutions of different concentrations. Indicated values are means (*n* = 4) ± SD. * *p* < 0.05, ** *p* < 0.01 and *** *p* < 0.001

As second approach to increase the lipophilicity of semaglutide, hydrophobic ion pairing (HIP) was investigated. Ethyl lauroyl arginate was chosen as cationic counter ion since it is an approved food additive and generally regarded as safe (GRAS). Previous studies successfully proved enhancement of lipophilicity for small molecules [39]. A charge ratio of 1:1 (peptide:surfactant) was applied as in general, highest precipitation efficiencies with equimolar charge ratios for peptides similar to semaglutide were observed [40, 41]. pH 8.0 for a 5 mg/mL semaglutide in 0.001 M NaOH with ethyl lauroyl arginate in a 1:1 charge ratio, results in ionization of anionic amino acids as the pH is above the isoelectric point (IEP) of semaglutide (IEP_Semaglutide_: 5.4). In contrast, higher pH values (pH 11.2 and pH 12.6) did not result in higher precipitation efficiencies suggesting interference of increasing ionic strength in the solution with the ion pair formation (Fig. 4b) [42]. Ionic bond formation between semaglutide and guanidine head group of the surfactant was confirmed by the IR spectra of the native surfactant and the hydrophobic ion pair, indicated by a strong reduction of the guanidine double bond signal in the complex (Fig. 4c). For reference (poly)peptides only low precipitation efficiencies < 50% were achieved utilizing the same method (0.0 ± 1.7% and 39.1 ± 2.2% for 14.4 kDa reference polypeptide and 66.4 kDa reference polypeptide, respectively).

### Increase in lipophilicity

Increase in lipophilicity characterized by the log D_n−octanol/water_ of formed complexes correlated nicely with the trend already observed within precipitation efficiency studies, where semaglutide HIP formed at pH 8 exhibited highest lipophilicity (Fig. 4d). This might be explained on the one hand by the formation of stronger ionic bonds at a pH being clearly above the IEP of semaglutide, but retaining the positive charge on the counter ion. On the other hand, the increasing amount of sodium ions present during complex formation was already shown to destabilize HIP [23, 41]. Compared to the native peptide a 2.4 × 10^4^-fold improvement in lipophilicity was achieved, similar to HIP formed with other GLP-1 analogues like exenatide [9, 10]. An even higher improvement in lipophilicity was obtained for semaglutide in RM with log D_octanol/water_ above 2. These results show that semaglutide is stronger retained within RM compared to HIP, hence allowing a higher fraction of the encapsulated semaglutide to reach the absorption membrane in intact form [43].

**Fig. 5:**
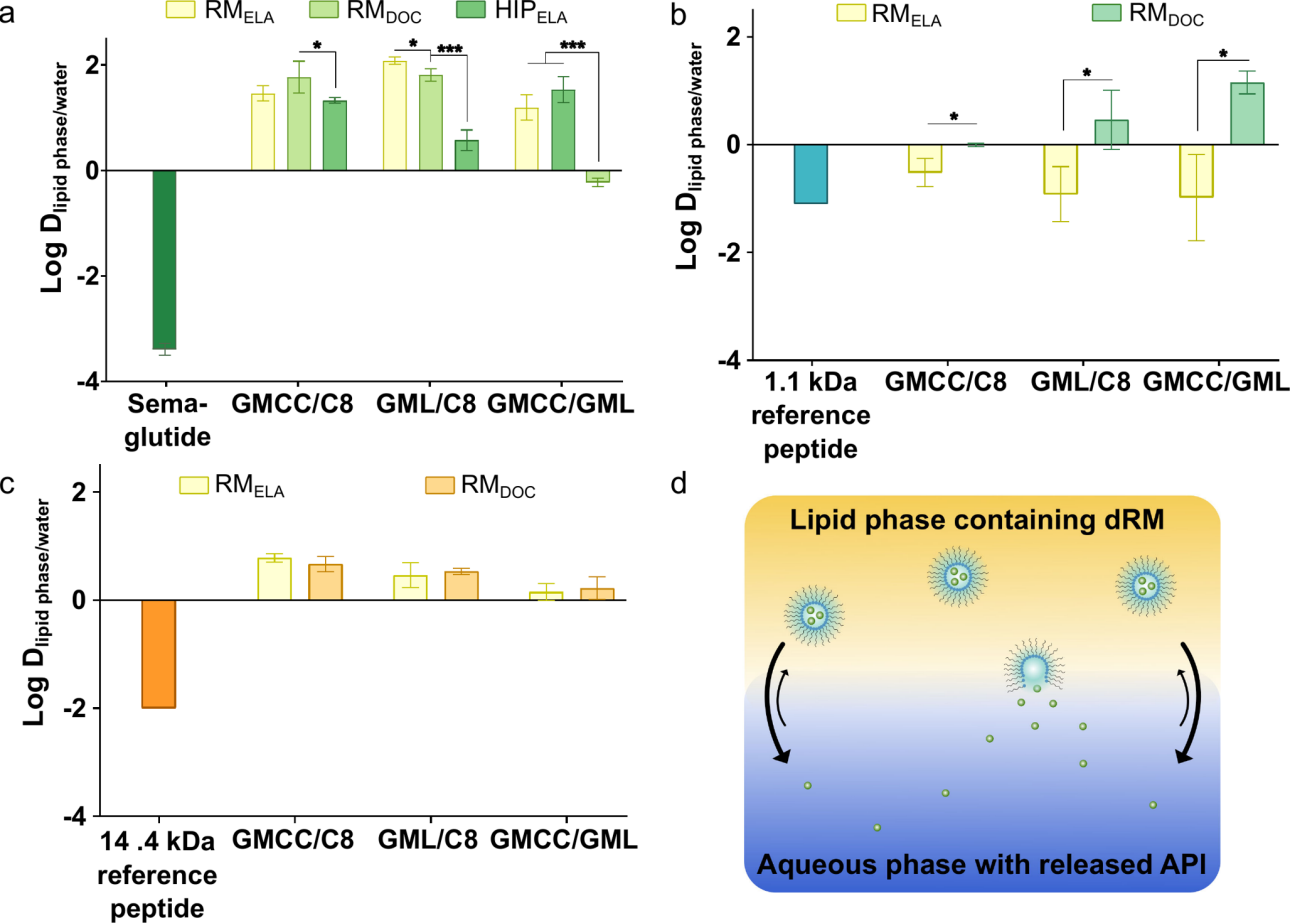
Distribution coefficient log D_lipid phase/water_ of **a** semaglutide, **b** 1.1 kDa reference peptide and **c** 14.4 kDa reference protein within reverse micelles. **d** Visualization of principle of distribution coefficient for peptides solubilized in lipid phase by dry reverse micelles (RM). Indicated values are means (*n* = 4) ± SD. API: active pharmaceutical ingredient, 1 kDa reference peptide, 14 kDa: reference protein, GMCC/C8: glycerol monocaprylocaprate:caprylic acid 1:4 (m/m), GMCC/GML: glycerol monocaprylocaprate:glycerol monolinoleaate 1:4 (m/m), GML/C8: glycerol monolinoleate:caprylic acid 1:4 (m/m). * *p* < 0.05 and *** *p* < 0.001

In comparison to hydrophobic ion pairs a higher log D_lipid phase/water_ for semaglutide was obtained with RM. The higher lipophilicity proves strong retention of the hydrophilic peptide within the polar cavity of the reverse micelles. In addition, the theoretically higher number of surfactant molecules per peptide leads to higher lipophilicity compared to the charge dependent formation of ion pairs [44–46]. Nevertheless, stabilizing effect of reverse micelles by opposite charge between the peptide and the surfactant is indicated by a higher lipophilicity of reverse micelles formed with ELA compared to docusate, as semaglutide bears a higher number of anionic than cationic moieties. This trend could also be confirmed for the 1.1 kDa reference peptide (colistin), as enhanced lipophilicity was achieved with docusate, whereas ethyl lauroyl arginate resulted in almost no increase in lipophilicity (Fig. 5b). It suggests stabilization of RM by opposing charges between surfactant and semaglutide, whereas same charges result in repulsive forces, favoring the release of the (poly)peptide to the aqueous phase. The effect of charge was lower for the 14.4 kDa reference polypeptide with a more balanced ratio of anionic and cationic moieties that causes only minor differences between docusate and ELA regarding lipophilicity (Fig. 5b, c). In direct comparison to HIP, RM showed at least a 2-fold improvement in lipophilicity confirming the capability of RM to improve lipophilicity of semaglutide. In contrast to previously published HIP with the 14.4 kDa reference polypeptide an up to 600-fold improvement in lipophilicity in RM compared to the native peptide and a 7.5-fold improvement with HIP was achieved [47].

### Development of SEDDS

Based on solubility and log D values described above, glycerol monocaprylocaprate (GMCC): caprylic acid (C8) 1:4 (m/m) was utilized as lipophilic phase for the development of unloaded SEDDS. Both of the lipid phases also provide permeation enhancing properties seeming beneficial to further boost performance of SEDDS [48]. As emulsifier, commonly utilized and widespread pharmaceutical excipient, PEG-35 castor oil (PEG-35 CO), was combined with the lipid phase GMCC/C8 1:4 (m/m) or RM in a 1:1 (m/m) ratio in order to obtain sufficient self-emulsification. Lower fractions of emulsifier were found to result in droplet sizes above 400 nm, PDI above 0.4 or aggregation over time (data not shown). Time for complete emulsification of all SEDDS was clearly below one minute demonstrating high self-emulsifying properties (Table 3). Immediately after emulsification droplet sizes were in a range of 75 nm to 326 nm, providing favorable permeation properties [49]. A monodisperse size distribution was achieved indicated by a polydispersity index (PDI) below 0.3.

**Table 3:** Droplet size, polydispersity index (PDI), zeta potential and emulsification time of placebo SEDDS after 1:100 (v/v) dilution with purified water after emulsification (T0) and 4 h of incubation (T4)

Formulation	T0			T4			Emulsification time [min]
	Droplet size [nm]	PDI	ζ-Potential [mV]	Droplet size [nm]	PDI	ζ-Potential [mV]	
SEDDS 1 (RM_ELA_)	119.29 ± 9.44	0.29 ± 0.03	29.89 ± 1.04	113.45 ± 0.61	0.30 ± 0.00	27.89 ± 3.21	0.63 ± 0.03
SEDDS 2 (RM_DOC_)	134.59 ± 12.27	0.27 ± 0.04	-56.83 ± 3.57	122.04 ± 9.73	0.27 ± 0.01	-47.30 ± 4.01	0.62 ± 0.27
SEDDS 3 (HIP_ELA_)	226.66 ± 28.52	0.24 ± 0.01	-8.96 ± 0.34	202.38 ± 11.74	0.31 ± 0.08	-8.81 ± 0.99	0.19 ± 0.03

Indicated values are means (*n* ≥ 3) ± SD

#### SEDDS containing RM and HIP

For loaded formulations, SEDDS 2 exhibited smallest droplets around 50 nm demonstrating beneficial effect of docusate for the formation of nanodroplets. Nevertheless, both RM containing formulations showed stability over 4 h without any significant changes in droplet size and PDI in water (Table 4). Zeta potential of the formulations was characteristic for the type of surfactant used for RM formation with a strongly positive value for ELA containing SEDDS, whereas clearly negative values were shown for docusate containing formulations. This indicates migration of surfactant molecules that are not involved in RM formation to the oil-water interface of formed nanodroplets after contact with water. The effect seemed to be more pronounced for docusate containing SEDDS compared to ethyl lauroyl arginate containing SEDDS since a pronounced drop in zeta potential was observed for SEDDS 2 after 4 h. In contrast, SEDDS containing HIP exhibited a zeta potential around zero, confirming strong ionic interactions of charged head group of ELA with semaglutide (Table 4). In the gastric environment slight increases in droplet size were observed for SEDDS 2 and SEDDS 3 still remaining below 350 nm, whereas SEDDS 1 remained unchanged over 4 h. Due to the high ionic strength of FaSSGF resulting in a conductivity above 3000 µS, zeta potential measurements were not feasible for samples diluted with FaSSGF, even after additional 1:9 (v/v) dilution with water. Direct comparison of SEDDS containing RM with SEDDS comprising HIP showed higher stability of RM containing formulations potentially assisted by the presence of docusate or ethyl lauroyl arginate, whereas SEDDS containing HIP lack this additional stabilization, characterized by an increase in droplet size of almost 100 nm after 4 h of incubation.

**Table 4:** Droplet size, polydispersity index (PDI) and zeta potential of semaglutide loaded SEDDS after 1:100 (v/v) dilution with purified water or fasted-state simulated gastric fluid (FaSSGF) pH 1.6 after emulsification (T0) and 4 h of incubation (T4)

Media	Formulation	T0			T4		
		Droplet size [nm]	PDI	ζ-Potential [mV]	Droplet size [nm]	PDI	ζ-Potential [mV]
Water	SEDDS 1 (RM_ELA_)	292.66 ± 74.18	0.33 ± 0.04	31.7 ± 2.7	294.79 ± 27.99	0.27 ± 0.08	35.7 ± 2.3
	SEDDS 2 (RM_DOC_)	48.96 ± 11.02	0.24 ± 0.01	-19.9 ± 1.4	55.25 ± 21.08	0.20 ± 0.04	-45.5 ± 2.21
	SEDDS 3 (HIP_ELA_)	264.27 ± 19.58	0.30 ± 0.03	-0.6 ± 0.8	361.31 ± 24.78	0.24 ± 0.10	1.9 ± 0.2
FaSSGF	SEDDS 1 (RM_ELA_)	222.38 ± 8.23	0.33 ± 0.01	-	227.81 ± 12.65	0.32 ± 0.02	-
	SEDDS 2 (RM_DOC_)	260.83 ± 14.16	0.11 ± 0.04	-	335.19 ± 15.92	0.16 ± 0.10	-
	SEDDS 3 (HIP_ELA_)	302.87 ± 19.70	0.14 ± 0.08	-	423.67 ± 13.03	0.31 ± 0.03	-

Indicated values are means (*n* ≥ 3) ± SD

**Fig. 6:**
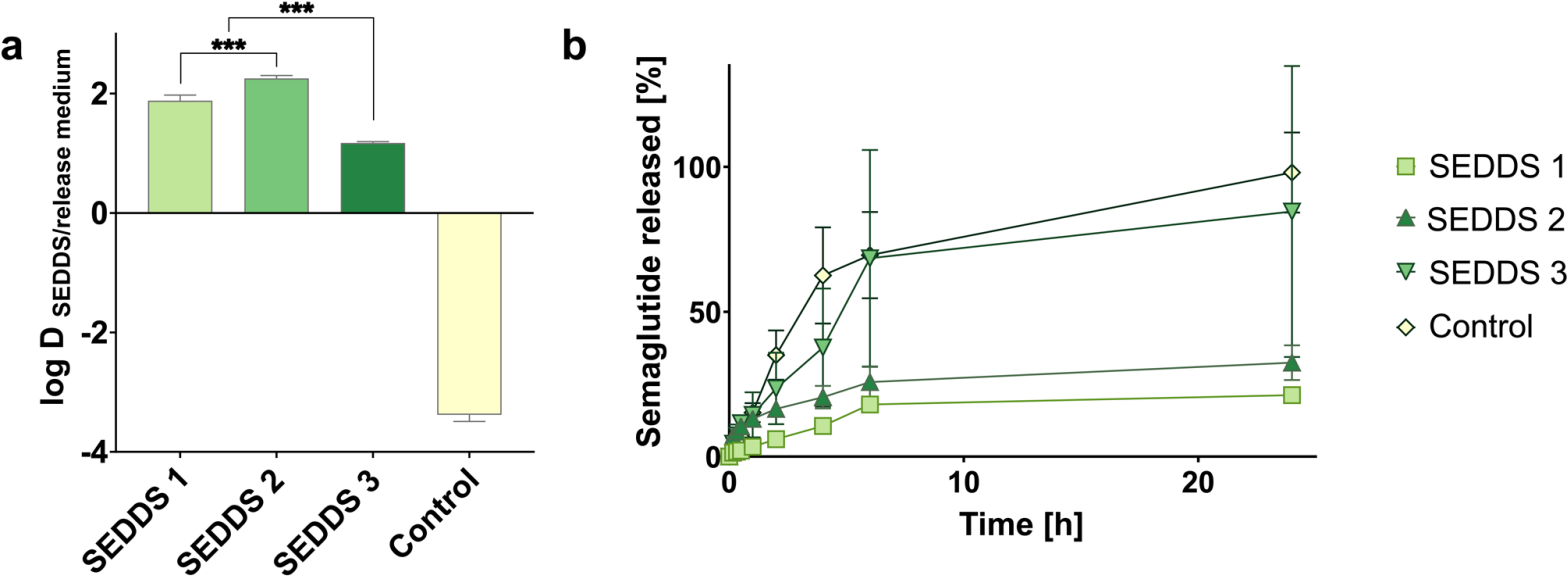
Distribution coefficient log D_SEDDS/water_ of **a** semaglutide loaded in SEDDS by RM (SEDDS 1 and SEDDS 2) or HIP (SEDDS 3). Release of **b** semaglutide from SEDDS. Indicated values are means (*n* = 4) ± SD. *** *p* < 0.001

Lowest semaglutide payload within the formulations was detected for SEDDS 3 containing HIP (6.3 ± 0.2 mg/mL; 12.6 ± 0.4% EE) which might be a result of a log D around 1 for the HIP, thus resulting in insufficient enhancement of lipophilicity for incorporation of the peptide in SEDDS. In contrast, RM containing SEDDS exhibited payloads of 24.2 ± 8.8 mg/mL (96.8 ± 9.7% EE) and 16.3 ± 6.0 mg/mL (65.2 ± 2.0% EE) for SEDDS 1 (RM_ELA_) and SEDDS 2 (RM_DOC_), respectively. Log D value of RM containing SEDDS 1 and SEDDS 2 showed higher lipophilicity enhancement of semaglutide compared to HIP (Fig. 6a). As the release of HIP is the same as for the control, semaglutide seems to be immediately released, which would also fit to the log D_SEDDS/Release medium_ (Fig. 6b). Based on these results, SEDDS containing semaglutide loaded RM can retain semaglutide within nanodroplets sufficiently long to reach the target membrane in intact form.

#### Toxicological studies

**Fig. 7:**
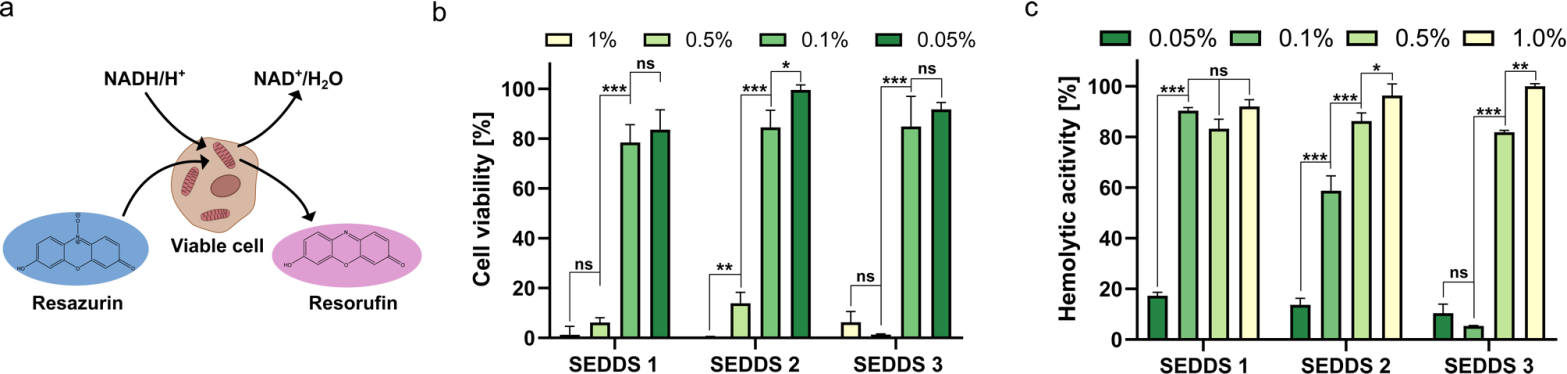
**a** Schematic drawing of reduction of resazurin by viable cells to resorufin used for detection of **b** cell viability of Caco-2 cells. **c** Hemolytic activity of SEDDS. Indicated values are means (*n* = 4) ± SD. ns: not significant, * *p* < 0.05, ** *p* < 0.01 and *** *p* < 0.001

Cell viability studies on Caco-2 cells indicated cytocompatibility for concentrations up to 0.1% (v/v) of RM and HIP containing SEDDS by a cell survival rate ≥ 75% (Fig. 7b) which is considered as nontoxic for Caco-2 cells. In general, SEDDS loaded with RM containing ELA showed slightly higher toxicity. The higher toxicity results most likely from the overall higher amount of the cationic surfactants within SEDDS 1 compared to SEDDS 3 where the cationic surfactant is complexed in ion pairs and only to a minor extent available at the surface of nanodroplets. Ethyl lauroyl arginate is used as food additive (E 243) because of its antimicrobial properties and was recently shown to efficiently remove oral biofilms [50, 51]. These properties likely result in the observed higher toxicity of ethyl lauroyl arginate comprising RM formulation compared to docusate. Nevertheless, no pronounced difference in cell viability between RM and HIP containing SEDDS could be observed indicating similar tolerability of both types of formulations. Furthermore, similar results as generally known for SEDDS regarding cell compatibility were obtained [52, 53]. Based on the cellular viability all formulations displayed almost identical cytotoxic effects, even though they bear clearly different surface charges. However, different mechanisms might be responsible for the toxicity observed depending on the surface charge. Cationic nanoparticles were shown to mainly cause membrane toxicity, whereas anionic nanoparticles show mostly intracellular toxicity [54, 55]. Membrane disrupting effects can be observed by hemolysis assay. Especially for cationic surfactants high interaction with cellular membranes were shown. This is most likely a result of the presence of cationic charged ELA on the surface of the nanodroplets [56, 57]. Thus, cationic RM formed with ELA show high hemolysis already at 0.1% (Fig. 7c). In contrast, RM formed with anionic docusate showed a hemolytic activity below 15% at the lowest investigated concentration, that is in accordance with previous studies [14].

Hemolytic activity of SEDDS can also be linked to the surfactants and the concentrations thereof as well as the charge density present on the surface of formulations [58]. RM formulations contain a higher total amount of surfactant molecules compared to HIP comprising SEDDS. As a result of the higher concentration of ionic surfactants, both RM containing SEDDS (SEDDS 1 and 2) resulted in more pronounced hemolytic activity compared to the formulation containing HIP (SEDDS 3).

#### Caco-2 permeation studies

**Fig. 8:**
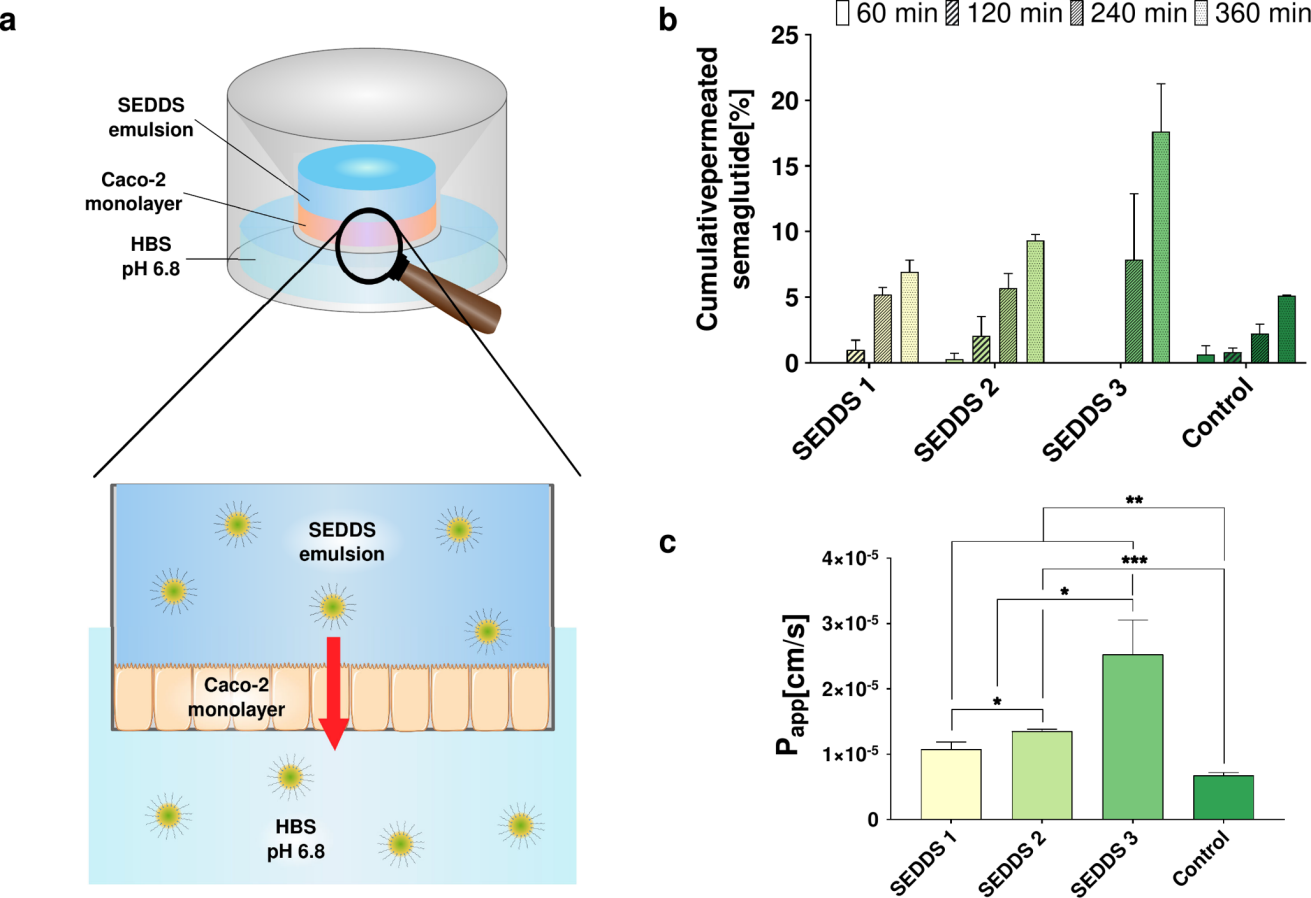
**a** Schematic drawing of experimental set up utilized for cell permeation studies. **b** Amount of SEDDS permeated trough Caco-2 monolayer over a period of 6 h and **c** calculated apparent permeability coefficient (P_app_). Indicated values are means (*n* = 4) ± SD. * *p* < 0.05, ** *p* < 0.01 and *** *p* < 0.001

In contrast to the semaglutide solution, a significant enhancement in permeation of semaglutide could be observed for the cationic SEDDS 1 and the anionic SEDDS 2, indicating high permeability of both reverse micelle containing formulations (Fig. 8a). The significantly higher permeability of SEDDS 2 compared to cationic SEDDS 1 could be explained by the markedly smaller droplet size, allowing a more efficient transport of SEDDS [59, 60]. This result indicates enhanced permeability for nanocarriers with droplet sizes below 100 nm as already observed within previous studies [61–64]. As SEDDS are known to enter the cellular entity by fusogenic processes and even more important endocytosis, a disruption of the nanoemulsion after fusion with the epithelial membrane is expected leading to a release of RM into the intracellular compartment. In comparison to formulations SEDDS 1 and SEDDS 2 comprising RM, even significantly higher permeability was shown for SEDDS 3 (Fig. 8b). This higher permeation shown for SEDDS 3 is likely linked to a premature release of HIP from these SEDDS (Fig. 6). However, under in vivo conditions the presence of numerous other counter ions will cause dissociation of the complex [41]. Furthermore, the released peptide will be degraded by peptidases. Overall, developed SEDDS displayed a high permeability through Caco-2 monolayer indicated by an apparent permeability coefficient (Papp) above 10^− 5^ cm/s [65]. In contrast, native semaglutide showed a significantly lower P_app_ value and under in vivo conditions would be rapidly degraded by proteolytic enzymes in the gastrointestinal tract. This demonstrates the benefit of SEDDS for the formulation of semaglutide known to protect the drug against proteolytic degradation [66, 67] and increasing the permeability of semaglutide.

## Conclusion

The present study compares two different strategies to incorporate semaglutide into SEDDS. In contrast to the current gold standard for enhancement of lipophilicity of peptides by HIP, a substantial improvement in the payload of semaglutide within SEDDS was achieved by dissolution of the GLP-1 analogue in lipophilic solvents by RM. Moreover, the incorporation of semaglutide within RM enables interaction of a higher number of surfactant molecules with the peptide, boosting the lipophilicity and enhancing the stability of the formed complexes. This raises the amount of dissolved semaglutide within SEDDS and results in a decreased release of the drug in biomimetic fluids. Because of this, a higher concentration of semaglutide is able to reach the absorption membrane in intact form. In particular, for opposite charges between the surfactant head group and the predominant charge on the peptide, higher lipophilic character of the complexes was achieved. For Caco-2 permeability of SEDDS, a significantly higher semaglutide permeation for all developed formulations demonstrated the beneficial effect of lipophilic complexes for semaglutide delivery.

Based on this study, combinatory approaches of strong ionic interactions between surfactants forming the reverse micellar system and semaglutide are suggested to further increase membrane permeation of semaglutide. In a broader perspective, this study demonstrated the applicability of SEDDS for the oral delivery of semaglutide and may pave the way to the development of even more efficient drug delivery systems for semaglutide to the benefit of diabetes type-2 or obesity patients.

## Electronic supplementary material

Below is the link to the electronic supplementary material.

Supplementary Material 1

## Data Availability

The authors confirm that the data supporting the findings of this study are available within the article.

## Abbreviations

API: Active pharmaceutical ingredient
BSA: Bovine serum albumin
C8: Caprylic acid
Caco-2: Human colon adenocarcinoma cell line
CO: Castor oil
COL: Colistin
DLS: Dynamic light scattering
DOC: Docusate
EE: Encapsulation efficiency
ELA: Ethyl lauroyl arginate
FaSSGF: Fasted-state simulated gastric fluid
FCS: Fetal calve serum
FTIR: Fourier-transformed infrared spectroscopy
GLP-1: Glucagon like peptide-1
GMCC: Glycerol monocaprylocaprate
GML: Glycerol monolinoleate
HBS: HEPES buffered saline
HEPES: 4-(2-Hydroxyethyl)-1-piperazineethanesulfonic acid
HIP: Hydrophobic ion pair(s)
HPLC: High pressure liquid chromatography
LR: Lumogen Red
LY: Lumogen Yellow
LYS: Lysozyme
NC: Nanocarrier
P_app_: Apparent permeability coefficient
PBS: Phosphate buffered saline
PDI: Polydispersity index
PE: Precipitation efficiency
PEG: Polyethylene glycol
RCF: Relative centrifugal force
rCMC: Reverse critical micelle concentration
Red MEM: Red minimum essential medium
RM: Reverse micelles
RSD: Relative standard deviation
RT: Room temperature
SEDDS: Self-emulsifying drug delivery systems
TCNQ: 7:7:8:8-tetracyanodimethane
TEER: Transepithelial electronic resistance
UV: Ultraviolet
