# Supplementary material for: Design of self-emulsifying oral delivery systems for semaglutide: reverse micelles versus hydrophobic ion pairs
Source: Drug Deliv Transl Res. 2024 Oct 19;15(6):2146–61. doi: 10.1007/s13346-024-01729-0 (PMC12037675; doi:10.1007/s13346-024-01729-0)
Supplement: Supplementary file 1 — Supplementary Material 1 [file 13346_2024_1729_MOESM1_ESM.docx]

**Supporting information: Design of self-emulsifying oral delivery systems for semaglutide: reverse micelles versus hydrophobic ion pairs**

Matthias Sandmeier^1,2^ ([0000-0002-6988-8015](https://orcid.org/0000-0002-6988-8015)), Fabricio Ricci^1,2^, Dennis To^1^ ([0009-0007-2159-7618](https://orcid.org/0009-0007-2159-7618)), Sera Lindner^1,2^, Daniel Stengel^1^, Michaela Schifferle^1^, Saadet Koz^1^, Andreas Bernkop-Schnürch^1^* ([0000-0003-4187-8277](https://orcid.org/0000-0003-4187-8277))

Table S1: Overview gradient applied for quantification of semaglutide by HPLC.

| Time [min] | Eluent A [%] | Eluent B [%] |
| --- | --- | --- |
| 0.0 | 50 | 50 |
| 2.0 | 50 | 50 |
| 6.0 | 30 | 70 |
| 6.1 | 50 | 50 |
| 10.0 | 50 | 50 |

Table S2: Overview gradient applied for quantification of bovine serum albumin by HPLC.

| Time [min] | Eluent A [%] | Eluent B [%] |
| --- | --- | --- |
| 0.0 | 80 | 20 |
| 1.0 | 80 | 20 |
| 7.5 | 40 | 60 |
| 8.0 | 80 | 20 |
| 12.0 | 80 | 20 |

Table S3: Overview gradient applied for quantification of colistin by HPLC.

| Time [min] | Eluent A [%] | Eluent B [%] |
| --- | --- | --- |
| 0.0 | 70 | 30 |
| 5.0 | 50 | 50 |
| 6.0 | 70 | 30 |
| 12.0 | 70 | 30 |

Figure S1: Representative HPLC chromatogram for 250 mg/mL semaglutide solution in ethanol (EtOH).

Figure S2: Representative HPLC chromatogram for 250 mg/mL bovine serum albumin solution in water:EtOH 1:19 (v/v) + 0.1% trifluoracetic acid (TFA).

Figure S3: Representative HPLC chromatogram for 250 mg/mL lysozyme solution in methanol (MeOH).

Figure S4: Representative HPLC chromatogram for 250 mg/mL colistin solution in EtOH.

Table S4: Overview of the composition of fasted state simulated gastric fluid (FaSSGF).

|  | FaSSGF |
| --- | --- |
| Sodium chloride (NaCl) | 199.9 mg |
| Hydrochloric acid (HCl 1 M) | 2910.0 mg |
| 3F Powder™ | 6.0 mg |
| Water | ad 100 ml |
